# Revisiting Wittgenstein’s puzzle: hierarchical encoding and comparison facilitate learning of probabilistic relational categories

**DOI:** 10.3389/fpsyg.2015.00110

**Published:** 2015-02-10

**Authors:** Wookyoung Jung, John E. Hummel

**Affiliations:** Relational Perception and Thinking Laboratory, Department of Psychology, University of Illinois at Urbana-ChampaignChampaign, IL, USA

**Keywords:** relational category learning, family resemblance, relational invariants, polysemy hypothesis, comparison, hierarchical categories

## Abstract

Kittur et al. (2004, 2006) and Jung and Hummel (2011, 2014) showed that people have great difficulty learning relation-based categories with a probabilistic (i.e., family resemblance) structure, in which no single relation is shared by all members of a category. Yet acquisition of such categories is not strictly impossible: in all these studies, roughly half the participants eventually learned to criterion. What are these participants doing that the other half are not? We hypothesized that successful participants were those who divided the nominal categories into two or more sub-categories, each of which individually had a deterministic structure. We report three experiments testing this hypothesis: explicitly presenting participants with hierarchical (category and sub-category) structures facilitated the acquisition of otherwise probabilistic relational categories, but only when participants learned the subordinate-level (i.e., deterministic) categories prior to learning the nominal (i.e., probabilistic) categories and only when they were permitted to view multiple exemplars of the same category simultaneously. These findings suggest that one way to learn natural relational categories with a probabilistic structure [e.g., Wittgenstein’s (1953), category *game*, or even *mother*] is by learning deterministic subordinate-level concepts first and connecting them together under a common concept or label. They also add to the literature suggesting that comparison of multiple exemplars plays an instrumental role in relational learning.

## INTRODUCTION

One of the most generally accepted assumptions in the literature on categorization and category learning is that categories and exemplars are mentally represented as lists of features and that the process of assigning exemplars to categories is based on comparing their features (for reviews, see Murphy, 2002; Kittur et al., 2006). As pointed out by Barsalou (1983), Gentner (1983), Murphy and Medin (1985) and others, one limitation of this view is that many concepts and categories are based, not on the literal features of their exemplars, but on relations—either relations among an exemplar’s features (e.g., arranged in one way, the parts of a folding bed form a bed, but arranged in another, they form a couch; Biederman, 1987; Hummel and Biederman, 1992) or relations between the exemplar and other objects in the world (e.g., the category *conduit* is defined by a relation between the conduit and the thing it carries; *barrier* is defined by the relation between the barrier, the thing to which it blocks access and the thing deprived of that access; even *mother* is defined by a relation between the mother and her child. Such concepts include both *role-governed categories* (Markman and Stilwell, 2001), such as *friend*, *mother*, *conduit* and *key*, which are defined by an object’s role relative to another object and full-blown, multi-role schemas, such as *transaction* (see, e.g., Gentner, 2005; Gentner and Kurtz, 2005; see also Hummel and Holyoak, 2003). Relational categories may be more the rule than the exception: informal ratings by Asmuth and Gentner (2005) of the 100 highest-frequency nouns in the British National Corpus revealed that about half of these nouns refer to relational concepts. The distinction between relational and feature-based categories need not pose a problem for the study of category learning as long as relational and featural categories are learned in similar ways. But if they are learned in different ways, then little or nothing we know about the acquisition of feature-based categories will necessarily apply to the case of relational concepts and categories.

Consider the well-known *prototype effects* in category learning (effects so robust they led Murphy, 2002, to quip that any experiment that fails to show them is suspect). One of the most basic of these effects is that participants are capable of learning categories with a *family resemblance* structure—that is, a structure in which every member of a category shares more features with the prototype of its own category than it does with the prototype of the contrasting category, but no single feature is shared by all members of the category. As noted by Kittur et al. (2004, 2006), this effect has always been demonstrated using categories defined by their exemplars’ features. These researchers wondered whether they could also demonstrate prototype effects in categories defined, not by the exemplars’ features, but by the relations among those features. In Kittur et al.’s (2004, 2006) experiments each exemplar was a two-part “object” consisting of an octagon and a square. In the prototype of one category, the octagon was *larger* than the square, *darker* than the square, *above* the square (in the picture plane) and *in front of* the square; in the prototype of the other category, the octagon was *smaller*, *lighter*, *below,* and *behind* the square. In a design typical of experiments demonstrating prototype effects, the categories had a family resemblance structure, such that each exemplar possessed three relations typical of its own prototype and one relation typical of the opposite prototype and no relation was shared by all members of either category.

Consistent with the hypothesis that relational categories are *not* learned in the same way as feature-based categories, Kittur et al. (2004, 2006); see also (Jung and Hummel, 2014) found that people have great difficulty learning relational categories with a probabilistic (i.e., family resemblance) structure. Their findings are consistent with the hypothesis that people learn relational concepts by a process of *intersection discovery*. Numerous researchers have proposed that relational concepts are represented as *schemas^1^*: relational structures that specify the properties of a concept or category exemplar and the relations among those properties and between the concept and other concepts (e.g., Gentner, 1983; Murphy and Medin, 1985; Holland et al., 1986; Keil, 1989; Barsalou, 1993; Ross and Spalding, 1994). In turn, it has been proposed (e.g., Gick and Holyoak, 1980, 1983; Hummel and Holyoak, 2003) that schemas are learned by a process of structural alignment (i.e., analogical mapping; see Hummel and Holyoak, 2003) followed by *intersection discovery*, in which a schema is learned from examples by keeping what the examples have in common and disregarding details on which they differ (see also Doumas et al., 2008). Alignment and intersection discovery are useful because they can reveal relational generalities that might otherwise remain implicit in the mental representation of the individual exemplars (see Doumas et al., 2008). However, intersection discovery fails catastrophically with probabilistic categories, in which the intersection is the empty set: by definition, the intersection is that which is common to all exemplars; in a probabilistic category structure, nothing is common to all exemplars. The findings of Kittur et al. (2004, 2006) are consistent with this account of their participants’ failure to learn their category structures.

Jung and Hummel (2014) extended the Kittur et al. (2004, 2006) findings by exploring the conditions under which probabilistic relational categories can be rendered learnable. Our logic was as follows: if the intersection discovery account of how we learn relational categories is correct, then any task that encourages participants to discover a relation that remains invariant over members of a category (and which differs between categories) ought to make otherwise probabilistic relational categories learnable. In order to test this hypothesis we created categories with a logical structure identical to that of Kittur et al. (2004, 2006): every exemplar consisted of a circle and a square, one of which was *larger than* the other, one of which was *darker*, one of which was *in front* the other, and one of which was *above* the other. In the prototype of category A, the circle was *larger*, *darker*, *above* and *in front of* the square; in B it was *smaller*, *lighter*, *below,* and *behind*. Each exemplar of either A or B shared three relations with its prototype, and no relation was constant across all members of a category. (In the Kittur et al. (2004, 2006) studies, the category structures were identical except that an octagon was used in place of the circle.) All we manipulated between participants was the instructions participants were given, and thus the task they were nominally performing.

In the “categorize” condition of Jung and Hummel (2014), Experiment 1, participants were instructed to categorize each exemplar as either an A or a B. In the “who’s winning?” condition, participants were instructed to determine whether the circle or the square was “winning.” Importantly, the tasks were otherwise completely isomorphic: any exemplar a participant in the “winning” condition would correctly classify as “the circle is winning” (by pressing the A key), a participant in the categorize condition would correctly classify as a member of category A (by pressing the A key); and any exemplar correctly classified as “the square is winning” (by pressing the B key) would correctly be categorized as a member of category B (by pressing the B key). We hypothesized that the “who’s winning” task—but not the categorize task—would encourage participants to discover a higher-order relation that remained invariant over members of a category (namely, which part, the circle or square, was in more “winning” roles of the four relations) and thus render the categories learnable. In several experiments, the results were exactly as predicted: participants given the “who’s winning” task learned to criterion much faster (and a much higher proportion of them reached criterion at all) than participants given the categorize task, even though the correct response to each exemplar was exactly the same across the tasks. This result is consistent with Kittur et al.’s (2004, 2006) interpretation of their findings in terms of participants invoking the psychological mechanisms responsible for schema induction (by intersection discovery) when faced with the task of learning a relational category structure. Specifically, the invariant participants appear to be learning in the “who’s winning” condition is something like, “The circle [or square] has more points, so it wins.” In the case of this invariant, it does not matter *which* relations give rise to the points; it only matters which shape has more of them. As a result, this learning procedure is robust to the variation in the individual relations giving rise to the “points.”

Although participants in the “who’s winning” condition learned much faster and more reliably than those in the categorize condition, as noted previously, roughly half the participants even in the categorize condition eventually learned to correctly classify the exemplars. Our primary motivation in the current study was to investigate what makes the probabilistic relational categories learnable in those participants that do manage to learn them. On the strictest interpretation of the intersection discovery hypothesis, this ought to be impossible: the intersection is always the empty set, so the categories should never be learnable by anyone. How do those participants who learn the categories manage to do so?

### POLYSEMY, HIERARCHICAL CATEGORIES, AND PROBABILISTIC RELATIONAL CATEGORIES

One possibility, suggested by Lakoff (1987), is that putatively probabilistic relational categories may in fact be *polysemous*. Consider for example, the category *mother*. Mother is a relational category (since a person’s membership in the category is defined by her relationship to her child), and although it may, at first, seem to be deterministic, there are in fact different kinds of mothers: birth mothers and adoptive mothers; caring and neglectful mothers; loving and abusive mothers, etc. The result is that no single relation (either genetic, care-based or emotional) necessarily characterizes every kind of mother. That is, *mother* is polysemous: a single label that refers to similar but nonetheless different categories of relationships. This possibility suggests a solution to the problem of learning probabilistic relational categories: rather than learning that all the exemplars belong to a single (probabilistic) category, perhaps it is easier to learn multiple sub-categories (each of which is individually deterministic), which are polysemous, in the sense of sharing a single label or name. Accordingly, we reasoned that the participants in the Kittur et al. (2004, 2006) and Jung and Hummel studies who managed to learn to criterion may have done so by treating the categories they were learning as polysemous: perhaps they somehow discovered subordinate-level categories that were deterministic by virtue of one or two relations remaining invariant, and then learned to classify those sub-categories with a common label (as elaborated shortly).

## THE CURRENT EXPERIMENTS

The current experiments tested three hypotheses about factors that might help people to learn otherwise probabilistic relational concepts. Experiment 1 tested the hypothesis that learning putatively probabilistic relational categories (like *mother*) can be facilitated by rendering such categories polysemous, that is, by training participants to learn deterministic sub-categories (i.e., “subordinate-level” categorizations) concurrently with the probabilistic category labels (i.e., “basic-level” categorizations). This experiment also tested the hypothesis that *comparison*—specifically, having the opportunity to explicitly compare the exemplars of the subordinate-level categories—would facilitate subordinate-level category learning. Comparison is thought to play a central role in schema induction (e.g., Gick and Holyoak, 1980, 1983; Gentner, 1983; Hummel and Holyoak, 2003) and relational learning (e.g., Doumas et al., 2008), and numerous studies have demonstrated the facilitatory effect of comparison on the learning of relational concepts (e.g., Hammer et al., 2008, 2009; Namy and Clepper, 2010; Augier and Thibaut, 2013; Kok et al., 2013; Kurtz et al., 2013; Carvalho and Goldstone, 2014; Guo et al., 2014). The results of Experiment 1 demonstrated that subordinate-level category learning facilitated participants’ learning of our probabilistic relational category structures, but only when participants were also allowed to compare multiple exemplars of a category to one another on each trial.

Experiment 2 extended Experiment 1 by investigating the necessity of the concurrent subordinate- and basic-level learning. In this experiment, participants were trained to classify exemplars at the probabilistic basic level before the deterministic subordinate level and learning did not improve relative to training on a basic-level-only baseline. Experiment 2 also investigated the effect of subordinate-level comparison without subordinate-level category learning. That is, it investigated whether giving learners the ability to compare two exemplars that would have belonged to the same subordinate-level category (and thus shared two invariant relations) during basic-level classification—but without explicit subordinate-level categorization— would improve basic-level learning relative to a one-exemplar baseline. It did not, suggesting that comparison, by itself, may not facilitate learning probabilistic relational categories.

Experiment 3 tested the hypothesis that presenting the (deterministic) prototype of each category alongside each (probabilistic) exemplar during training would facilitate learning. This manipulation is analogous to explicit instruction (e.g., in a classroom setting) that although the exemplars are probabilistic in the relations they possess, they nonetheless derive from a deterministic underlying category structure. The results suggest that this procedure, like the subordinate-before-basic procedure of Experiment 1, facilitated participants’ learning. This experiment also tested two additional variants of the comparison hypothesis tested in Experiment 1 and provided weak support for that hypothesis.

**FIGURE 1:**
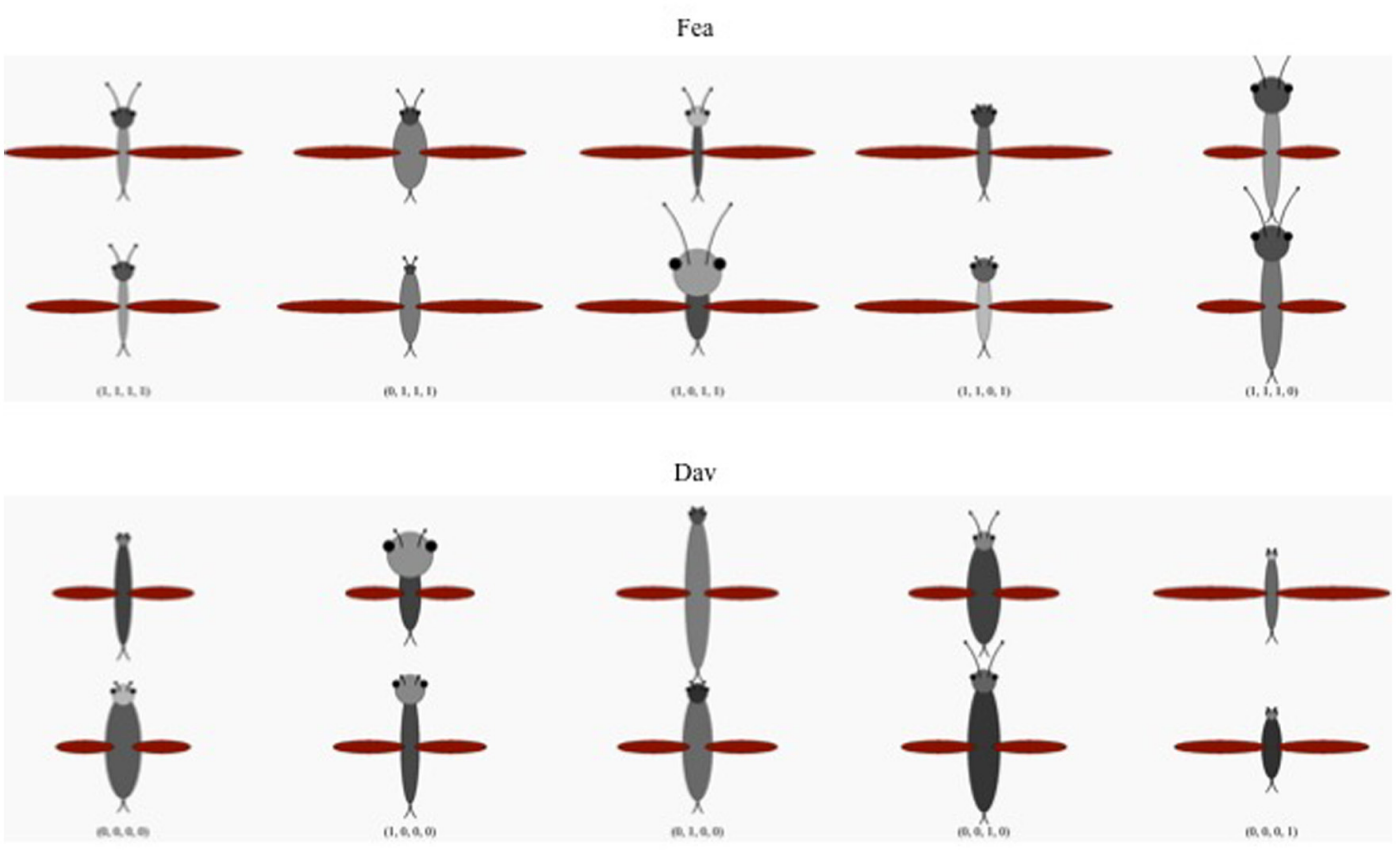
**Examples of the stimuli used in all the experiments.** The top two rows depict basic-level category *Fea*; the bottom two rows depict basic-level category *Dav*. The left most column shows two examples of each category prototype: Fea (1111) and Dav (0000). Columns 2…5 depict two examples each of the categories’ specific exemplar classes. Columns correspond to the exception relation defining that class. For example, exemplars in column 2 (0111 and 1000) differ from their respective prototypes in the relative width of the bugs’ heads and bodies (r1). Examples of a prototype or an exemplar class differ from one another in their metric properties (e.g., head width) but share categorical relations (e.g., whether the head is wider or narrower than the body). The figure shows two randomly selected examples of each prototype or exemplar class, out of an open-ended set of such examples (with each example differing from the others in its class in terms of its precise metric properties). See text for details.

An additional purpose of the current experiments was to replicate the basic difficulty-of-probabilistic-relational-category learning effect with new stimulus materials. Kittur et al. (2004, 2006) used stimuli composed of octagons and squares, and Jung and Hummel (2014), (Experiments 1…3) used stimuli composed of circles and squares. The current experiment used fictional “bugs” as stimuli (**Figure 1**). The purpose of using these new stimuli was simply to demonstrate whether the same effects obtain with very different (arguably, more natural) stimulus materials. Like the stimuli in the previous experiments, the categories used in the current experiments were defined by the relations among their exemplars’ parts, and individual relations were probabilistically related to category membership across exemplars.

### CATEGORY STRUCTURES

The categories used in these experiments were fictional “bug species” defined by the relations between the bugs’ head, body, wings, and antennae. As shown in **Figure 1** and **Table 1**, the prototype of the category (species) “Fea” [1, 1, 1, 1] had a head *wider* and *darker* than its body (relations r1 and r2; the first two 1’s in the vector), antennae *longer* than its head (r3) and wings *longer* than its body (r4). The prototypical Dav [0, 0, 0, 0] had the opposite relations, with its body *wider* and *darker* than its head (r1 and r2), antennae *shorter* than its head (r3) and wings *shorter* than its body (r4).

**Table 1:** The prototype and exemplar class for each species.

Fea species (1111)	Dav species (0000)
0111	1000
1011	0100
1101	0010
1110	0001

Any exemplar of Fea or Dav shared three relations with its own prototype and one with the prototype of the opposite category (**Table 1**). In other words, the formal category structures used were isomorphic to those used by Kittur et al. (2004, 2006) and Jung and Hummel (2014). All members of an exemplar class (where a class corresponds to one of the eight binary codes in **Table 1**) share exactly the same defining relations (e.g., all members of 0111 have a heads that are narrower and darker than their bodies, antennae longer than their heads and wings longer than their bodies) but differ from one another in the exact numerical dimensions and darknesses of their heads, wings, antennae, and bodies. That is, although relationally identical to one another, members of an exemplar class are featurally different from one another. Stimuli were generated by the computer while the subject performed the experiment, randomly choosing the metric values of the bugs’ parts to be consistent with the defining relations. As such, it is unlikely that any given subject would see exactly the same bug more than once during the experiment.

In Experiment 1, participants learned to classify the bugs at a subordinate level (Cim Fea [first two exemplar classes in Column1 of **Table 1**], Kei Fea [last two exemplar classes in Column 1], Sko Dav [first two exemplar classes, Column 2] or Lif Dav [last two exemplar classes, Column 2]). In Experiment 2, participants learned to classify the bugs at both the basic level (Fea vs. Dav) and at the subordinate level. In Experiment 3, participants learned each exemplar class as its own unique subordinate-level category (Kei Fea, Bai Fea, Wou Fea, or Cim Fea for the Fea species; Haw Dav, Ang Dav, Sko Dav, or Lif Dav for the Dav species). The basic level categories were probabilistic, in the sense that each relation was diagnostic of category membership 75% of the time, but no single relation was fully diagnostic. However, each subordinate-level category had two fully deterministic relations. For example, in the two exemplars of Cim Fea, [1101] and [1110], relations r1 and r2 both deterministically take the value 1; and in the two exemplars of Sko Dav, [0010] and [0001], both take the value 0. As such, Fea and Dav are polysemous, with deterministic subordinate level categories.

## EXPERIMENT 1

Experiment 1 investigated the hypothesis that learning the categories’ deterministic subordinate-level labels would facilitate participants’ learning of their polysemous (probabilistic) basic-level labels. It also investigated the necessity of explicit subordinate-level comparison for the learning of the subordinate-level categories.

### METHOD

#### Participants

Forty five undergraduates enrolled at the University of Illinois participated in Experiment 1 for course credit.

#### Materials

Stimuli were line drawings of fictional bugs as described above. Subspecies of each species were made by grouping pairs of exemplars according to shared relations: Kei Fea = [0, 1, 1, 1] and [1, 0, 1, 1,], and Cim Fea = [1, 1, 0, 1] and [1, 1, 1, 0]; Sko Dav = [1, 0, 0, 0] and [0, 1, 0, 0], and Lif Dav = [0, 0, 1, 0], and [0, 0, 0, 1]. Eight trials per block were presented in the *subordinate-level with comparison* condition, and 16 trials per block were presented in the *subordinate-level without comparison* and *basic baseline* conditions. Each exemplar was presented in a random order once (*subordinate-level with comparison* condition) or twice (*subordinate-level without comparison* and *basic baseline* conditions) per block. There were only half as many trials per block in the *subordinate-level with comparison* condition as in the other two conditions because each trial of *subordinate-level with comparison* presented two versions of each exemplar at a time, whereas the other conditions presented only one exemplar per trial.

#### Design

The experiment used a three-condition (*subordinate-level with comparison* vs. *subordinate-level without comparison* vs. *basic baseline)* between-subjects design.

#### Procedure

All conditions consisted of two or more blocks of training trials followed by two blocks of transfer trials. The training phase of the experiment differed across conditions, as described above. During this phase of the experiment, participants received accuracy feedback on each trial.

In the *subordinate-level with comparison* condition, each trial of the training phase simultaneously presented two exemplars belonging to the same subordinate-level category. Participants identified the stimuli at the subordinate level (i.e., as Cim Fea, Kei Fea, Sko Dav or Lif Dav) by clicking one of four boxes depicting the relevant subordinate- and basic-level names under the two bugs. This response was followed by accuracy feedback. See the Appendix for figures depicting the participants’ task in each condition of each experiment reported here.

In the *subordinate-level without comparison* condition (Figure A2 in the Appendix), each trial depicted a single stimulus (rather than a pair), but otherwise the procedure was identical to that in the *subordinate-level with comparison* condition. In the *basic baseline* condition, each trial depicted a single bug, which the participant classified at the basic level only (Figure A3 in the Appendix). In all three conditions, this training phase was followed by a transfer phase.

The training phase lasted 40 blocks (320 trials for the two *subordinate-level with comparison* condition and 640 trials for the other conditions) or until the participant responded correctly on at least 87.5% (7/8 or 14/16) of the trials for two consecutive blocks, whichever came first. The transfer phase was the same across all conditions. All participants classified the bugs at the basic level only and received no accuracy feedback. 16 trials were presented per block, with each exemplar presented in a random order once per block. Each exemplar remained on the screen until the participant responded. At the end of the experiment participants were queried about strategies they used during the experiment.

### RESULTS

#### Trials to criterion

Most of the participants (12 of 15) reached criterion in *subordinate-level with comparison*, whereas only 1 of 15 reached criterion in *subordinate-level without comparison* and none reached criterion in *basic baseline*. A chi-square test of independence showed that trials-to-criterion differed reliably across conditions [χ2 (2, *N* = 45) = 25.187, *p* < 0.001].

**FIGURE 2:**
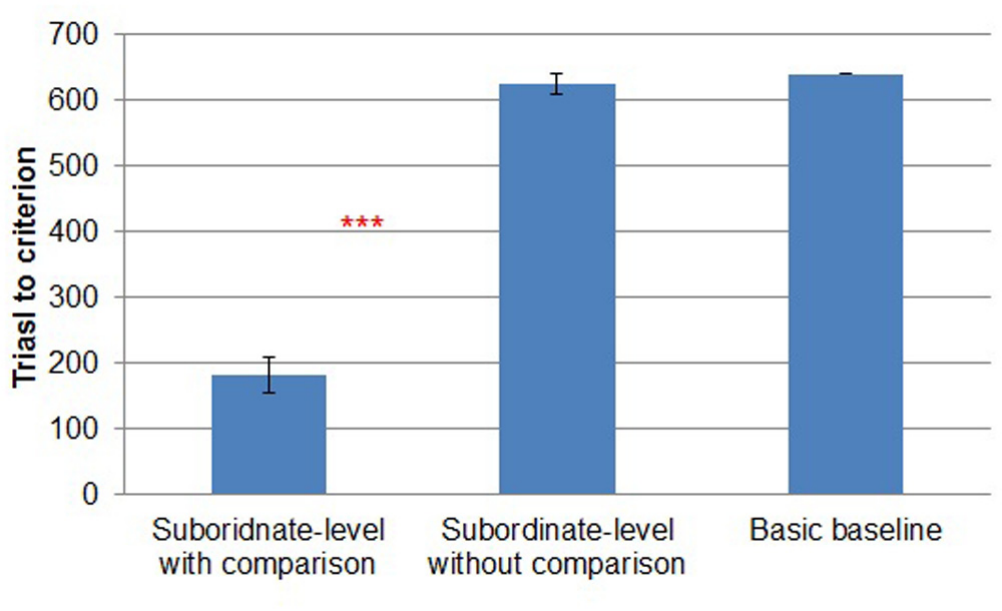
**Trials to criterion by study condition in Experiment 1.** Error bars represent SEs. ***p < 0.001.

In addition to the chi-square test, in all three experiments we performed a more conservative test of our hypothesis (i.e., more favorable to the null hypothesis) by comparing trials to criterion across conditions (**Figure 2**). (Rather than converting each subject to a binary, *did reach criterion* vs. *did not reach criterion* as in the chi-square test, the differences in trials to criterion preserve metric differences between participants’ performance.) We made this test even more conservative by treating those participants who failed to reach criterion as though they had reached criterion in the last block of learning. There was a reliable difference between *subordinate-level with comparison* (*M* = 182, *SD* = 108) and *subordinate-level without comparison* (*M* = 625, *SD* = 58) [*t*(28) = -14.014, *p* < 0.001]. The performance in *basic baseline* was the worst overall (*M* = 640, *SD* = 0).

#### Study phase accuracy

**FIGURE 3:**
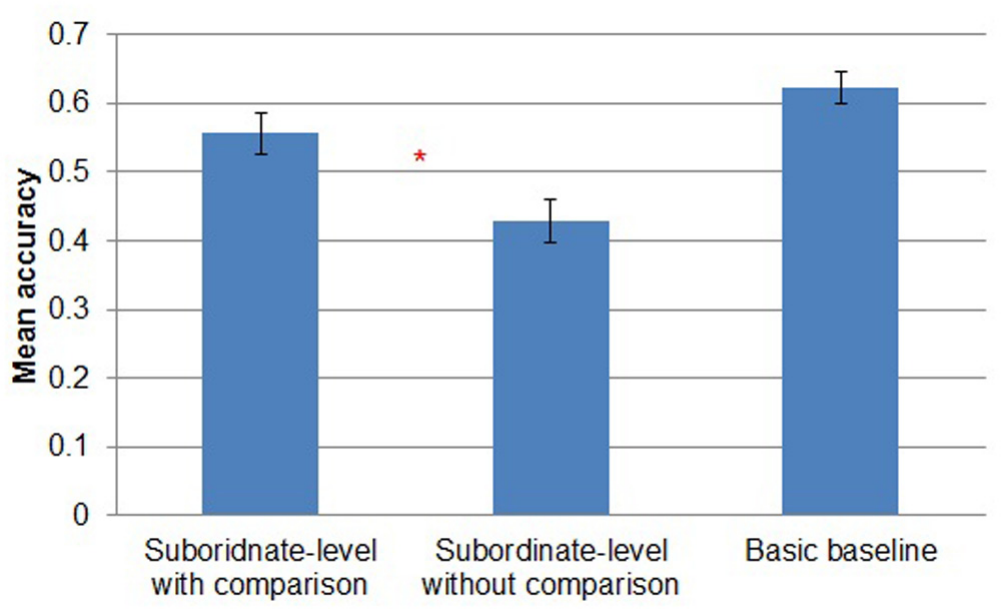
**Mean accuracy by study condition in Experiment 1.** Error bars represent SEs. *p < 0.05.

First, we report accuracy of subordinate-level classification (Kei Fea, Cim Fea, Sko Dav or Lif Dav) in the *subordinate-level with comparison* and *subordinate-level without comparison* conditions and accuracy of basic-level classification (Fea or Dav) in the *basic baseline* condition. Participants in *subordinate-level with comparison* (*M* = 0.56, *SD* = 0.12) were more accurate than participants in *subordinate-level without comparison* (*M* = 0.43, *SD* = 0.12) [*t*(28) = 2.928, *p* < 0.01]. Participants in *basic baseline* were the most accurate (*M* = 0.62, *SD* = 0.09; **Figure 3**). However, chance performance in the two subordinate-level conditions was 0.25 whereas chance in the *basic baseline* condition was 0.5, so it is difficult to compare study phase accuracy directly across these conditions. If we correct for chance performance by subtracting each participant’s mean accuracy by chance performance in the condition, then mean corrected accuracy is 0.31 in *subordinate with comparison condition*, 0.18 in *subordinate without comparison* and 0.12 in *basic baseline*. (Of course, this correction has no effect on the results of the *t*-tests.)

#### Transfer phase accuracy

**FIGURE 4:**
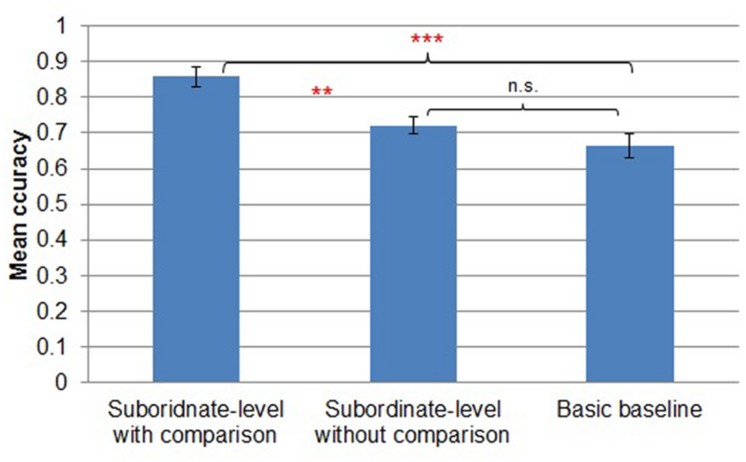
**Mean accuracy by transfer condition in Experiment 1.** Error bars represent SEs. **p < 0.01, ***p < 0.001.

A three-way (*subordinate-level with comparison* vs. *subordinate-level without comparison* vs. *basic baseline*) between-subjects ANOVA revealed main effects of task [*F*(2,44) = 11.880, MSE = 0.149, *p* < 0.001; **Figure 4**]. Participants in the *subordinate-level with comparison* condition (*M* = 0.86, *SD* = 0.11) showed reliably more accurate performance during transfer than participants in the *subordinate-level without comparison* (*M* = 0.72, *SD* = 0.09; Tukey’s HSD, *p* < 0.01) and *basic baseline* conditions (*M* = 0.67, *SD* = 0.13; Tukey’s HSD, *p* < 0.001). There was no reliable difference between transfer in the *subordinate-level without comparison* and *basic baseline* conditions (Tukey’s HSD, *p* = 0.36).

### DISCUSSION

Both in terms of trials to criterion during learning and in terms of accuracy of basic-level classification during transfer, training participants to classify stimuli at a deterministic subordinate level and allowing them to explicitly compare multiple exemplars of a subordinate-level category to one another (the *subordinate-level with comparison* condition) improved category learning relative to simply training the stimuli at the basic level only (the *basic baseline* condition) and relative to simply training the stimuli at the subordinate level without the opportunity to compare them (the *subordinate-level without comparison* condition). This finding suggests that, as hypothesized, rendering probabilistic relational categories polysemous (and thus deterministic at the subordinate level) makes them more learnable, but that this facilitatory effect of polysemy (at least in our data) depends on participants having the opportunity to compare members of the same subordinate-level category and thus observe which relations they have in common.

## EXPERIMENT 2

If deterministic subordinate-level learning is to facilitate probabilistic basic-level learning, then it seems necessary for the subordinate-level learning to temporally precede (or at least proceed at the same time as) the basic-level learning (see also Anderson, 1991; Love et al., 2004; Mathy et al., 2013) ^2^. Accordingly, in the subordinate-level conditions of Experiment 1, participants viewed pairs of exemplars from the same subordinate-level category on each trial and learned to classify the stimuli at the subordinate level before being required to transfer learning to the basic level. Experiment 2 investigated the necessity of the subordinate-before-basic learning order used in Experiment 1. In the *basic-level first with comparison* condition of Experiment 2, participants were trained to classify exemplars at the probabilistic basic level before classifying them at the deterministic subordinate level. This experiment also investigated the effect of subordinate-level comparison without subordinate-level category learning: in the *basic-level only with comparison* condition of this experiment, participants viewed pairs of exemplars that would have belonged to the same subordinate-level category, but only learned to classify them at the basic level. The *basic baseline* condition of Experiment 2 was identical to that condition of Experiment 1: on each trial, the participant viewed only a single exemplar and classified it only at the basic level.

### METHOD

#### Participants

Forty four undergraduates enrolled at the University of Illinois participated in the study for course credit. Participants were randomly assigned to one of three conditions.

#### Materials

The stimuli and category structures were identical to those used in Experiment 1.

#### Design

The experiment used a three condition (*basic-level first with comparison* vs. *basic-level only with comparison* vs. *basic baseline)* between-subjects design.

#### Procedure

All conditions consisted of two or more blocks of training trials followed by two blocks of transfer trials. The training phase of the experiment differed across conditions, as described above. During this phase of the experiment, participants received accuracy feedback on each response made on each trial.

In the *basic-level first with comparison* condition (Figure A4 in the Appendix), each trial of the training phase simultaneously presented two exemplars. Participants identified the stimuli at the basic level by clicking on boxes under the bugs. This response was followed by accuracy feedback. Next, they re-identified the same bugs at the subordinate-level. Although Figure A4 depicts both the subordinate- and basic-level response boxes (as though they were on the screen simultaneously for the subject), in the experiment, the subordinate-level response boxes appeared on the screen only after the subject had made her basic-level response. In the *basic-level only with comparison* condition (Figure A5 in the Appendix), participants again viewed pairs of bugs belonging to the same subordinate-level species, but were given only the basic-level identification task. In the *basic baseline* control condition, bugs were presented one at a time in the center of the screen, asking participants to identify each bug at the basic level.

The transfer phase was the same across all conditions and identical to that of Experiment 1.

### RESULTS

#### Trials to criterion

**FIGURE 5:**
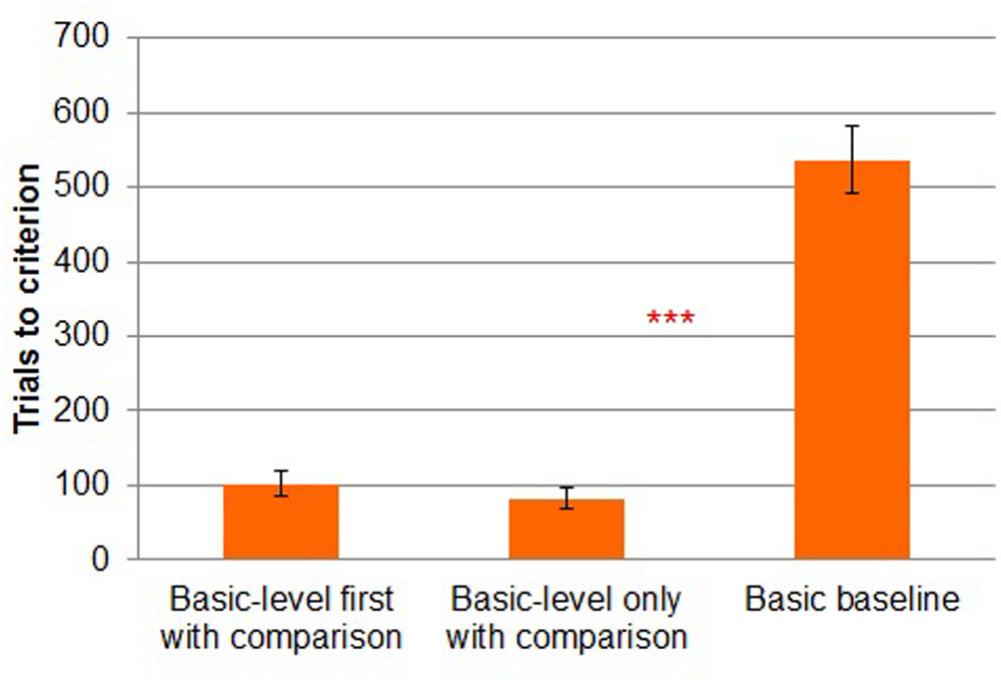
**Trials to criterion by study condition in Experiment 2.** Error bars represent SEs. ***p < 0.001.

All participants in the *basic-level first with comparison* and *basic-level only with comparison* conditions reached criterion, whereas only 50% of participants in the *basic baseline* condition reached criterion (**Figure 5**). A chi-square test revealed that the number of participants who reached criterion differed reliably by condition [χ2 (2, *N* = 44) = 18.904, *p* < 0.001].

A more conservative three-way (*basic-level first with comparison* vs. *basic-level only with comparison* vs. *basic baseline*) between-subjects ANOVA on trials to criterion revealed a main effect of task [*F*(2,41) = 78.511, MSE = 12482.691, *p* < 0.001]. As shown in **Figure 5**, participants in the *basic-level first with comparison* condition (*M* = 102, *SD* = 64) took reliably fewer trials to reach criterion than those in the *basic baseline* condition (*M* = 537, *SD* = 173; Tukey’s HSD, *p* < 0.001). Participants also reached criterion in fewer trials in *basic-level only with comparison* condition (*M* = 82, *SD* = 52) than in the *basic baseline condition* [Tukey’s HSD, *p* < 0.001]. However, the *basic-level first with comparison* and *basic-level only with comparison* conditions did not differ from one another reliable in terms of trials to criterion [Tukey’s HSD, *p* = 0.52].

#### Study phase accuracy

**FIGURE 6:**
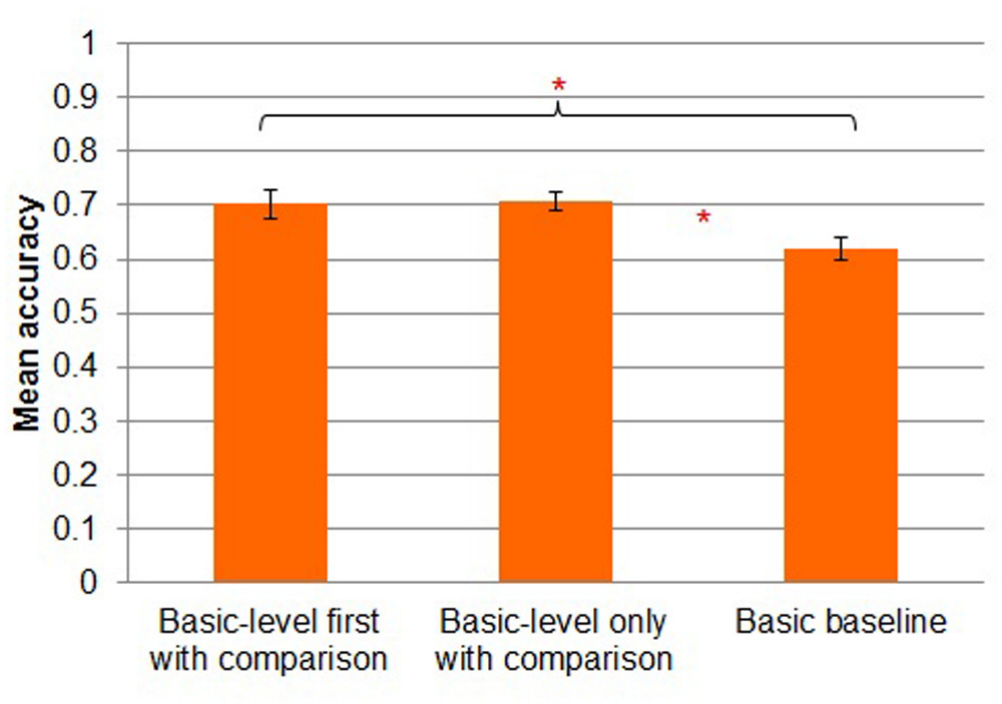
**Mean accuracy by study condition in Experiment 2.** Error bars represent SEs. *p < 0.05.

A three-way *(basic-level first with comparison* vs. *basic-level only with comparison* vs. *basic baseline*) between-subjects ANOVA on the data from the study phase revealed a main effect of condition [*F*(2,44) = 11.914, MSE = 0.145, *p* < 0.001; **Figure 6**]. Participants in the *basic-level first with comparison* condition were more accurate (*M* = 0.70, *SD* = 0.10) than participants in the *basic baseline* condition (*M* = 0.62, *SD* = 0.08; Tukey’s HSD, *p* < 0.05), as were participants in the *basic-level only with comparison* condition (*M* = 0.71, *SD* = 0.07; Tukey’s HSD, *p* < 0.05). Performance in the *basic-level first with comparison* condition was almost identical to performance in the *basic-level only with comparison* condition. (Tukey’s HSD, *p* = 0.99).

#### Transfer phase accuracy

**FIGURE 7:**
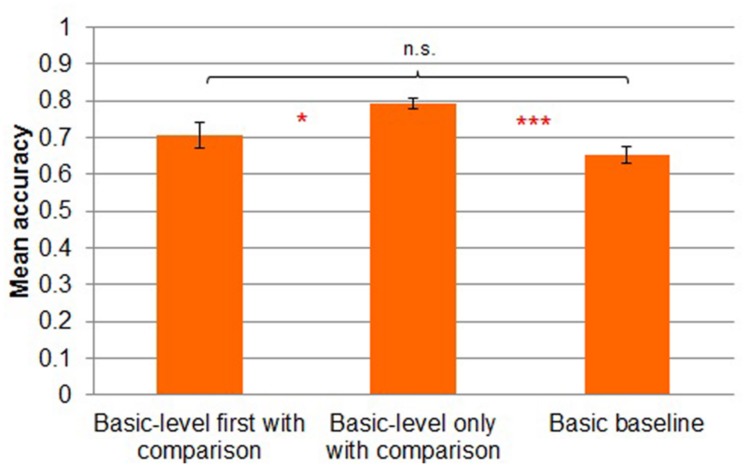
**Mean accuracy by transfer condition in Experiment 2.** Error bars represent SEs. *p < 0.05, ***p < 0.001.

A three-way (*basic-level first with comparison* vs. *basic-level only with comparison* vs. *basic baseline*) between-subjects ANOVA revealed main effects of task [*F*(2,41) = 9.298, MSE = 0.008, *p* < 0.001; **Figure 7**]. Participants in the *basic-level only with comparison* condition (*M* = 0.79, *SD* = 0.06) showed reliably more accurate performance during transfer than participants in the *basic-level first with comparison* condition (*M* = 0.71, *SD* = 0.13; Tukey’s HSD, *p* < 0.05) and in the *basic baseline* condition (*M* = 0.65, *SD* = 0.09; Tukey’s HSD, *p* < 0.001). There was no reliable difference between transfer in the *basic-level first with comparison* and *basic baseline* conditions (Tukey’s HSD, *p* = 0.32).

### DISCUSSION

The results of Experiment 2 replicate the facilitatory effect of comparison observed in Experiment 1 and are consistent with the hypothesis that adding a subordinate-level comparison does not facilitate learning unless it precedes basic-level learning. Participants in the *basic-level only with comparison* condition (who were able to compare exemplars of the same subordinate-level categories but who did not learn the subordinate-level labels) learned to criterion much faster than those in the *basic baseline* condition, but those in *basic-level first with comparison* (who did learn the subordinate-level label) did not learn faster than those in *basic-level only with comparison*. In fact, the trend, although not statistically reliable, was in the opposite direction. Likewise, in terms of transfer performance, participants in *basic-level only with comparison* categorized the stimuli more accurately than either those in *basic baseline* or those in *basic-level first with comparison*. When it follows the basic-level learning task, the subordinate-level task does not improve the rate of category learning and hinders transfer performance, relative to exemplar comparison alone.

## EXPERIMENT 3

Experiment 3 investigated whether exposure to the (deterministic) prototype of an otherwise probabilistic relational category, along with the members of that category, could facilitate participants’ learning of probabilistic relational categories. Our hypothesis was that comparing the exemplars to the prototype would help participants learn to categorize the stimuli in terms of prototype-plus-exception rules. For example, mapping the prototype [1, 1, 1, 1] to the exemplar [1, 0, 1, 1] might result in a schema that includes r1, r3, and r4, but lacks r2 (i.e., [1, -, 1, 1]). Whichever exemplar is compared to the prototype, the resulting schema will always produce one of the probabilistic category structures, minus the mismatching relation (i.e., in the case of Fea, [-, 1, 1, 1], [1, -, 1, 1], [1, 1, -, 1], or [1, 1, 1, -]).

Experiment 3 also differed from Experiments 1 and 2 in the design on the subordinate-level categories. In Experiments 1 and 2, each basic-level category had two subordinate-level categories, with the result that, within a subordinate-level category, two relations were deterministic and two were probabilistic. In Experiment 3, each basic-level category had four subordinate-level categories, with the result that, within a subordinate-level category, all four relations were deterministic.

On each trial in the *prototype* condition of Experiment 3 (Figure A6 in the Appendix), participants saw a prototype of a basic-level category (i.e., Fea or Dav) on the left of the screen and an exemplar of that species (i.e., an exemplar of one subordinate-level category) on the right of the screen. (Recall that the categories are defined in terms of the relations between the exemplars’ parts, not the precise metric values of those parts. As such, different instances of the prototype of a category will not be identical to one another, even though they will have all the same categorical relations between their parts.) Their task was to classify the prototype at the basic-level and the exemplar at its subordinate-level (e.g., Kei Fea, Bai Fea, Wou Fea, or Cim Fea). One again, although Figure A6 depicts both the basic- and subordinate-level response boxes, in the experiment, the subordinate-level response boxes appeared on the screen only after the subject had made her basic-level response. We hypothesized that providing the category prototype along with each exemplar of that category might provide participants with an explicit hierarchical structure that could facilitate category learning. If so, then each subordinate label would be associated with the relational difference between that exemplar and the prototype of its category (the *exception* relation): for example, Kei Fea (*narrower* head), Bai Fea (*lighter* head), Wou Fea (*shorter* antenna), and Cim Fea (*shorter* wing).

The *two different exemplars* condition (Figure A7 in the Appendix) tested whether presenting exemplars from the same basic-level category but different subordinate-level categories could facilitate learning. In this condition, each trial presented two different exemplars of the same basic-level category. After identifying the exemplars at the basic-level, participants also identified each exemplar at its own subordinate-level. (Again, the subordinate-level response boxes appeared on the screen after the subject made her basic-level response.) In this condition, exemplars were not paired within trials in order to correspond to systematic subordinate-level categories (i.e., they were not paired in a way that was likely to reveal invariant relations). Accordingly, the intersection discovery and polysemy hypotheses predict little or no facilitation in this condition relative to the *basic baseline* condition.

The *two same exemplars* condition of the current experiment (Figure A8 in the Appendix) presented two exemplars from the same subordinate level category on each trial. The task was to classify the stimuli first at the basic level and then at the subordinate level. (The subordinate-level response boxes appeared on the screen only after the subject made her basic-level response.) In this condition, the two exemplars had identical categorical relations between their parts but nonetheless differed in the metric properties of those parts.

In addition, there were two control conditions (Figure A9 in the Appendix). In the *subordinate baseline* condition, each trial presented a single bug and the task was to categorize it at the basic level first, followed by the subordinate level. The *basic baseline* condition was the same except that the participant’s only task was to classify the bug at the basic-level.

### METHOD

#### Participants

A total of 96 undergraduates enrolled at the University of Illinois participated in the study for course credit. Participants were randomly assigned to one of five conditions.

#### Materials

The same bug stimuli were used in this experiment as in Experiment 1 and 2. However, in this experiment, each exemplar, including the prototype, of each category was associated with a unique label: for the Fea species [1, 1, 1, 1] was the prototype (labeled simply “Fea”), Kei Fea = [0, 1, 1, 1], Bai Fea = [1, 0, 1, 1,], Wou Fea = [1, 1, 0, 1], and Cim Fea = [1, 1, 1, 0] served as the subordinates; for the Dav species [0, 0, 0, 0] was the prototype(labeled simply “Dav”), Haw Dav = [1, 0, 0, 0], Ang Dav = [0, 1, 0, 0], Sko Dav = [0, 0, 1, 0], and Lif Dav = [0, 0, 0, 1] were the subordinates.

#### Design

The experiment used a five-condition (*prototype* vs. t*wo different exemplars* vs. *two same exemplars* vs. *subordinate baseline* vs. *basic baseline)* between-subjects design.

#### Procedure

All conditions were provided two or more blocks of training trials consisting of basic and subordinate classification tasks (only the basic task was provided in *basic baseline*), followed by two blocks of transfer trials, as in the previous experiments. The training phase of the experiment differed across conditions, as described below. During training, participants received accuracy feedback on each trial. The transfer phase was the same across all conditions. Participants classified the bugs at the basic level only and they received no accuracy feedback.

In the *prototype* condition, participants were shown a prototype on the left side of the screen. They first categorized the prototype as Fea or Dav with a mouse click. Following this response, an exemplar of the same basic-level category appeared on the right of the screen (the prototype remained on the screen) and the participant classified it at its subordinate level with a button click.

In the *two different exemplars* condition, two different exemplars belonging to the same species, randomly chosen, were displayed simultaneously. Participants first classified both bugs at the basic level and then classified each at its own subordinate level.

The *two same exemplars* condition was identical to the *two different exemplars* condition, except that two exemplars came from the same subordinate level, so there was only one subordinate-level response.

In the *subordinate baseline* condition, the participant classified one bug per trial at both the basic and subordinate levels. In the *basic baseline* condition participants classified each bug at the basic level only.

In the *prototype*, *two different exemplars*, and *two same exemplars* conditions*,* each training block consisted of eight trials. In the *subordinate baseline*, and *basic baseline* conditions, each training block consisted of 16 trials. In all conditions, the transfer phase was identical to the learning phase of the *basic baseline* condition. There were two blocks of 16 trials each, with each exemplar presented in a random order once per block. The training phase lasted for 40 blocks (320 trials for *prototype*, *two different exemplars*, *and two same exemplars,* and 640 trials for *subordinate baseline*, and *basic baseline*) or until the participant responded correctly on at least 87.5% of the trials for two consecutive blocks. At the end of the experiment participants were queried about strategies they had used during the experiment.

### RESULTS

#### Trials to criterion

Only in the *prototype* condition did all participants reach criterion. 55% of participants reached criterion in the *two different exemplars* condition, 44% in the *two same different exemplars* condition, 16% in the *subordinate baseline* condition, and 68% in the basic baseline condition reached criterion. A chi-square test results showed that the proportions of participants who reached criterion differed reliably by condition [χ2 (4, *N* = 96) = 30.503, *p* < 0.001].

**FIGURE 8:**
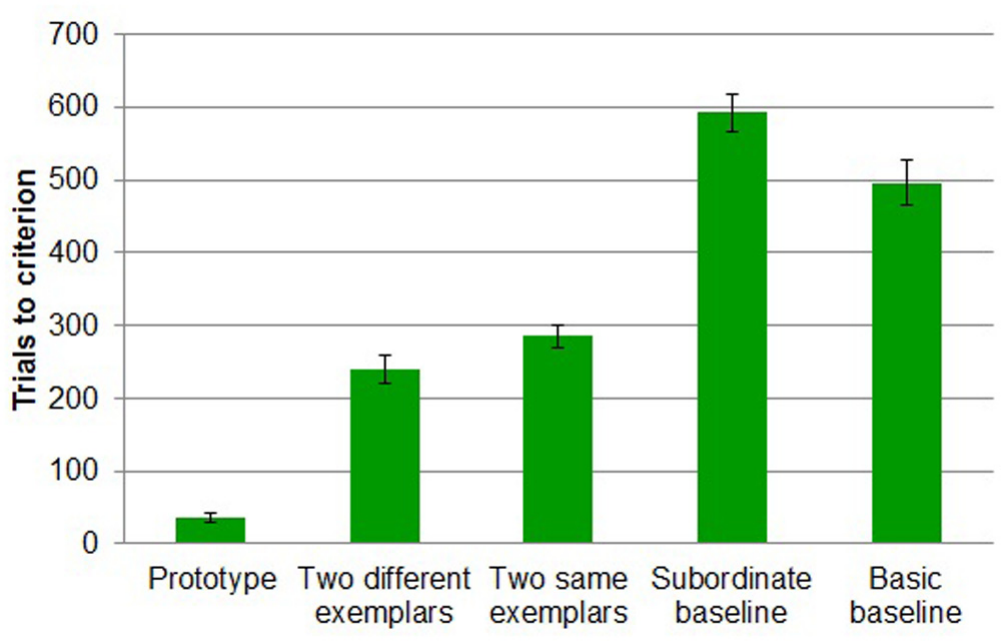
**Trials to criterion by study condition in Experiment 3.** Error bars represent SEs.

A more conservative five-way (*prototype* vs. t*wo different exemplars* vs. *two same exemplars* vs. *subordinate baseline* vs. *basic baseline*) between-subjects design ANOVA on trials to criterion revealed a main effect of task [*F*(4,91) = 107.139, MSE = 8776.459, *p* < 0.001; **Figure 8**]. As expected, participants reached criterion in fewer trials in *prototype* (*M* = 36, *SD* = 27) than in *two different exemplars* (*M* = 239, *SD* = 89; Tukey’s HSD, *p* < 0.001), *two same exemplars* (*M* = 285, *SD* = 69; Tukey’s HSD, *p* < 0.001), *subordinate baseline* (*M* = 592, *SD* = 113; Tukey’s HSD, *p* < 0.001), and *basic baseline* (*M* = 496, *SD* = 133; Tukey’s HSD, *p* < 0.001). Participants in *two different exemplars* task (*M* = 239, *SD* = 89) took reliably fewer trials to reach criterion than those in *subordinate baseline* (*M* = 592, *SD* = 113; Tukey’s HSD, *p* < 0.001) and those in *basic baseline* (*M* = 496, *SD* = 133; Tukey’s HSD, *p* < 0.001). Participants in *two same exemplars* (*M* = 285, *SD* = 69) also took reliably fewer trials to reach criterion than those in *subordinate baseline* (*M* = 592, *SD* = 113; Tukey’s HSD, *p* < 0.001) and those in *basic baseline* (*M* = 496, *SD* = 133; Tukey’s HSD, *p* < 0.001). Participants in *basic baseline* (*M* = 592, *SD* = 113) took reliably fewer trials to reach criterion than those in *subordinate baseline* (*M* = 496, *SD* = 133; Tukey’s HSD, *p* < 0.05).

#### Study phase accuracy

**FIGURE 9:**
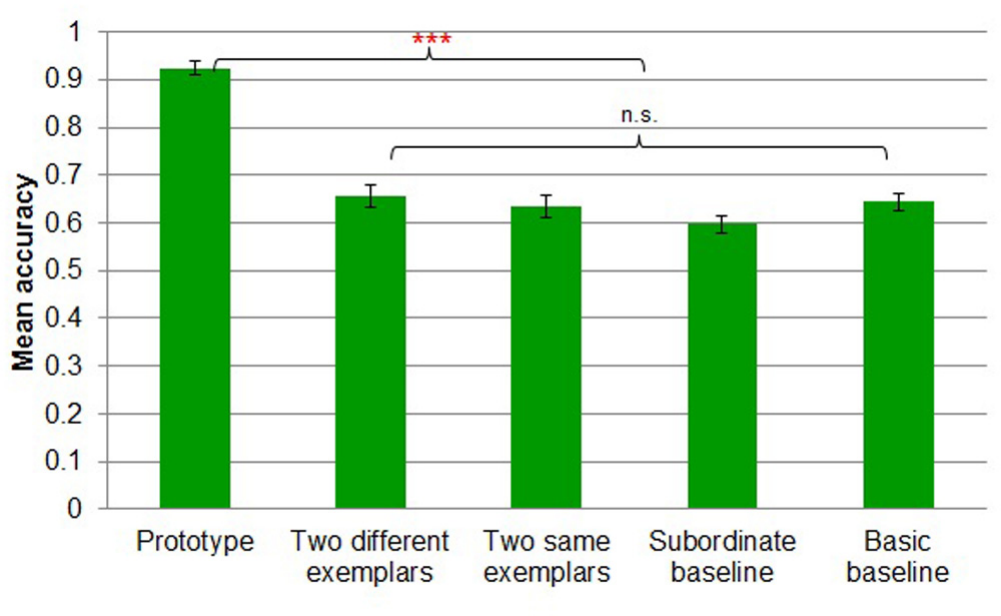
**Mean accuracy by study condition in Experiment 3.** Error bars represent SEs. ***p < 0.001.

A five-way *(prototype* vs. *two different exemplars* vs. *two same exemplars* vs. *subordinate baseline* vs. *basic baseline*) between-subjects ANOVA revealed a main effect of task [*F*(4,91) = 45.518, MSE = 0.008, *p* < 0.001; **Figure 9**]. Participants in the *prototype* condition performed more accurately than those in all other conditions. *Prototype* learners (*M* = 0.92, *SD* = 0.06) were likely to perform more accurately than *two different exemplars* learners (*M* = 0.66, *SD* = 0.11; Tukey’s HSD, *p* < 0.001), *two same exemplars* learners (*M* = 0.63, *SD* = 0.09; Tukey’s HSD, *p* < 0.001), *subordinate baseline* learners (*M* = 0.60, *SD* = 0.08; Tukey’s HSD, *p* < 0.001), and *basic baseline* learners (*M* = 0.64, *SD* = 0.08; Tukey’s HSD, *p* < 0.001). There were no other reliable differences between the conditions at the basic level during the study phase.

#### Transfer phase accuracy

**FIGURE 10:**
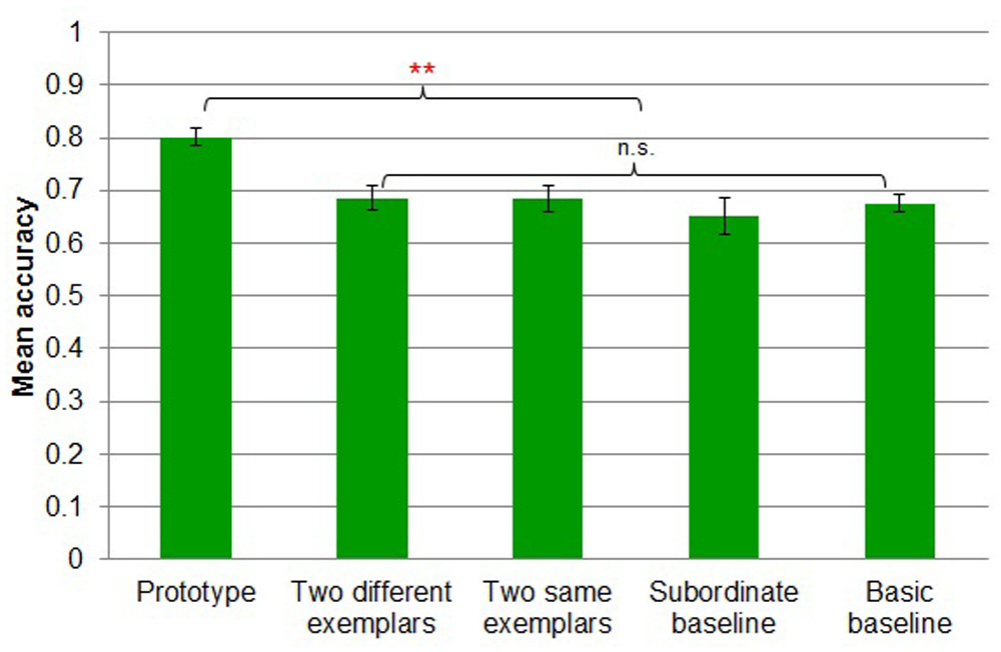
**Mean accuracy by transfer condition in Experiment 3.** Error bars represent SEs. **p < 0.01.

A five-way *(prototype* vs. t*wo different exemplars* vs. *two same exemplars* vs. *subordinate baseline* vs. *basic baseline*) between-subjects ANOVA revealed a main effect of task [*F*(4,91) = 5.943, MSE = 0.011, *p* < 0.001; **Figure 10**]. Participants in *prototype* (*M* = 0.80, *SD* = 0.07) showed reliably more accurate transfer performance than those in *two different exemplars* (*M* = 0.69, *SD* = 0.10; Tukey’s HSD, *p* < 0.01), *two same exemplars* (*M* = 0.68, *SD* = 0.11; Tukey’s HSD, *p* < 0.01), *subordinate baseline* (*M* = 0.65, *SD* = 0.16; Tukey’s HSD, *p* < 0.01) and *basic baseline* (*M* = 0.68, *SD* = 0.07; Tukey’s HSD, *p* < 0.01).

### DISCUSSION

Experiment 3 examined whether providing participants with prototypes of the basic-level categories would facilitate their learning of the exemplars of those categories. As expected, performance in the *prototype* condition exceeded performance in the other conditions. Participants in the *Prototype* condition were over 90% correct classifying the prototype during learning and 80% correct during transfer. Their accuracy during transfer demonstrates that, in addition to learning the prototypes, these participants also learned the exemplars. These results are consistent with the hypothesis that explicitly providing the prototype can help learners overcome the difficulties posed by the empty intersection problem, and are consistent with the hypothesis that explicitly providing the prototypes helps participants to learn the exemplars in a prototype-plus-exception fashion.

Consistent with the intersection discovery and polysemy hypotheses, randomly pairing different exemplars of the same basic-level categories during study (the *two different exemplars* condition) did not reliably improve learning over simply training one exemplar at a time (*basic baseline* or *subordinate baseline*). However, systematically pairing members of the same subordinate-level category (the *two same exemplars* condition) also failed to improve learning. Participants’ comparatively poor performance in the *two same exemplars* condition of this experiment represents a replication of the *basic level first* condition of Experiment 2.

## GENERAL DISCUSSION

Although people have great difficulty learning relational categories with a probabilistic structure, roughly half of the participants in previous studies on probabilistic relational category learning have eventually managed to do so (Kittur et al., 2004, 2006; Jung and Hummel, 2011, 2014). In the current study, three experiments tested the hypothesis that those participants who do manage to learn probabilistic relational categories do so by learning those categories as polysemous collections of deterministic subordinate-level categories. These experiments also investigated the role of explicit within-category comparison in this process, as well as the related hypothesis that explicitly exposing learners to the prototypes of probabilistic relational categories can help them to acquire these otherwise difficult-to-learn category structures.

Experiment 1 showed that, combined with the opportunity to explicitly compare members of the same (probabilistic) basic-level category, learning to categorize exemplars at a deterministic subordinate level facilitated subsequent learning of their probabilistic basic-level structures. However, without the opportunity to make these explicit comparisons, subordinate-level training was not sufficient to improve learning relative to basic-level category training. This finding is consistent with previous research demonstrating the role of explicit comparison in relational learning (Hammer et al., 2008, 2009; Namy and Clepper, 2010; Augier and Thibaut, 2013; Kok et al., 2013; Kurtz et al., 2013; Carvalho and Goldstone, 2014; Guo et al., 2014).

Experiment 2 demonstrated that training the basic- and subordinate-levels of classification concurrently (with the basic-level response preceding the subordinate-level response on a trial-by-trial basis) did not improve category learning relative to baseline (i.e., *basic-baseline*: training with the basic-level only). By contrast, comparison of multiple exemplars without subordinate-level training did facilitate category learning, albeit less without subordinate-level training than with subordinate-level training: although we did not conduct a between-experiment statistical test on the difference, the effect of subordinate-training-plus-comparison (86% mean accuracy, Experiment 1) was numerically greater than the effect of comparison-only (79% mean accuracy, Experiment 2). However, together, these experiments suggest that comparison of multiple exemplars has a greater impact on learning of probabilistic relational categories than does explicit subordinate-level training.

Experiment 3 demonstrated that explicitly training participants to classify the (deterministic) category prototypes helps them to learn to classify the individual (probabilistically related) exemplars. This effect is consistent with the hypothesis that explicit training with the prototype helps participants to learn the exemplars as specific exceptions to the otherwise deterministic category.

At first blush, the results of Experiments 1–3 appear to provide only weak support for the hypothesis that those participants (e.g., in Kittur et al., 2004; Jung and Hummel, 2011, 2014) who manage to learn probabilistic relational categories do so by first learning to categorize those exemplars as members of individually deterministic subordinate-level categories. Instead, comparison seems to play a much larger role in participants’ acquisition of our probabilistic relational bug categories, whether comparison of exemplars within the same subordinate-level category (the s*ubordinate with comparison* condition of Experiment 1 and the *basic only with comparison* condition of Experiment 2) or comparison of exemplars with their category prototype (the *prototype* condition of Experiment 3).

However, it is important to note that comparison, alone, did not facilitate learning: in particular, comparing randomly paired exemplars of the same category (the *two different exemplar*s condition of Experiment 3) did not improve learning relative to baseline. Instead, only those comparisons that made it possible for learners to discover relations that remain invariant, either over subordinate-level categories (Experiments 1 and 2) or over the prototype itself (Experiment 3), facilitated learning. As long as learners are provided with the opportunity to make these invariant-revealing comparisons, it appears to make little difference whether the resulting subordinate-level categories are explicitly labeled. But without the opportunity to make these invariant-revealing comparisons, simply forcing participants to learn names for the invariant-bearing subordinate-level categories (e.g., the *subordinate without comparison* condition of Experiment 1) seems to make little difference to learning.

Applying these lessons to the case of polysemous real-world relational categories, such as *mother*, is revealing. As children, we do not need to learn separate names for adoptive vs. birth mothers, or for abusive vs. loving mothers. Instead, as pointed out by the label *polysemous*, all these different concepts bear the same simple name, *mother*. However, the findings reported here suggest that acquiring these different (subordinate-level, invariant-bearing) *mother* concepts may well be facilitated by the opportunity to explicitly compare exemplars of these various kinds of mothers.

The question of why explicit comparisons proved so crucial in the experiments reported here cannot be answered by our current findings, but it is tempting to speculate that without such comparisons, the memory load of comparing an exemplar on the computer screen to exemplars stored in memory may simply be too great. With two systematically related exemplars (or an exemplar and a prototype) on the screen in front of a learner, discovering which relations they have in common becomes a matter of perception: the learner need only move her attention between the bugs a few times to observe the relations they share and then encode those relations into memory. But with only a single bug on the screen, or with two bugs that are not systematically related (as in the *two different exemplars* condition of Experiment 3), this kind of simple perceptual inspection cannot reveal which relations are diagnostic of (sub-)category membership: if there is only one bug on the screen, then it must be compared with other bugs in memory; and before the bug has been categorized, the learner cannot even know which of the many bugs in memory to which it ought to be compared. And although having two unsystematically related bugs on the screen together alleviates the memory problem (rendering the discovery of shared relations a perceptual problem), the shared relations so revealed are not guaranteed to be the same as the shared relations in the next pair of bugs from the same basic-level category. Although this explanation of our findings is intuitive, investigating it more systematically is beyond the scope of the current work.

In conclusion, the findings reported here—specifically, the finding that comparisons that systematically reveal relations that remain invariant over sub-classes of otherwise probabilistic relational categories—add to the growing body of evidence that learning relational categories depends on, or is at least greatly facilitated by, conditions that lead the learner to discover relational invariants that predict category membership.

As a final note, it interesting to wonder whether the findings reported here may provide an answer to Wittgenstein’s (1953) challenge to define the category *game*. The difficulty of defining *game* was taken by Wittgenstein (1953)—and by the majority of cognitive psychologists since then (see Murphy, 2002)—to mean that *game* is a family resemblance category with no necessary or sufficient features. But perhaps *game* is not a (feature-based) family resemblance category at all. Instead, it may be a collection of polysemous relational categories comprised of, for example, *board games*, *card games*, *war games*, *athletic games*, etc. And although it is impossible to find a single definition that captures all these different kinds of games (like it is impossible to find a single relation that characterizes all kinds of mothers), perhaps each individual kind of game is a deterministic relational category. If this account is correct, and game is a polysemous relational category, then Wittgenstein’s (1953) famous puzzle, *What is the definition of a game?* will turn out to have been a trick question.

## SUPPLEMENTARY MATERIAL

The Supplementary Material for this article can be found online at: http://www.frontiersin.org/journal/10.3389/fpsyg.2015.00110/abstract

## Conflict of Interest Statement

The authors declare that the research was conducted in the absence of any commercial or financial relationships that could be construed as a potential conflict of interest.

## Footnotes

1 For example a *songbird* schema might specify that a songbird eats seeds or insects, nests in trees, flies, and has wings and a beak (among other properties). In addition, it would specify that having wings makes it possible for the songbird to fly (i.e., explicitly specifying the relation between the properties *has wings* and the property *flies*) and that songbirds are members of the more general category birds (i.e., *specifying* the relation between *songbirds* and *birds*). Other kinds of schemas describe the critical relations characterizing other domains of knowledge (see, e.g., Gick and Holyoak, 1983).

2 In most circumstances, if *a* (e.g., subordinate-level learning) is to cause or facilitate *b* (basic-level learning), then *a* must precede (or at least operate concurrently with) *b*. In the context of basic- and subordinate-level category learning, this claim may appear at odds with the developmental literature, which shows that children learn terms for basic-level categories before they learn terms for subordinate-level categories (e.g., Rosch and Mervis, 1975; Johnson and Mervis, 1997). However, the order in which children lean names for things may not necessarily coincide exactly with the order in which concepts are acquired or even accessed on-line. Jolicoeur et al. (1984) showed that the *entry level* of object categorization—the level of first perceptual/conceptual access during object recognition—resides at a level below the basic level. For example, upon viewing a picture of a Boeing 747, one recognizes it as a *jumbo jet* (a subordinate-level categorization) before one recognizes it as an *airplane* (the basic-level categorization). In this sense, even the (possibly feature-based) concept *airplane* may be polysemous. Anderson (1991) and Love et al. (2004) have also noted that, depending on the structure of exemplars within categories, it is often advantageous to acquire subordinate-level concepts before higher-level ones, even when not explicitly directed to do so.
