# Supplementary material for: Revisiting Wittgenstein’s puzzle: hierarchical encoding and comparison facilitate learning of probabilistic relational categories
Source: Front Psychol. 2015 Feb 10;6:110. doi: 10.3389/fpsyg.2015.00110 (PMC4322609; doi:10.3389/fpsyg.2015.00110)
Supplement: Supplementary file 1 [file Presentation_1.PDF]

# Appendix

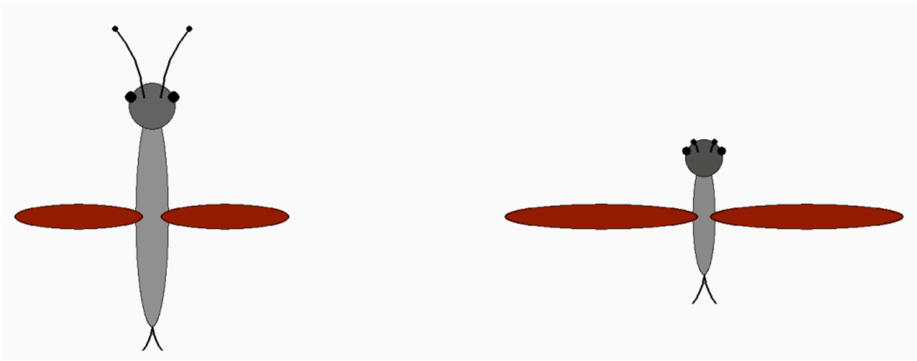

| |
|---|
| Cim Fea |
| Kei Fea |
| Sko Dav |
| Lif Dav |

Figure A1. The *subordinate-level with comparison* condition in Experiment 1.

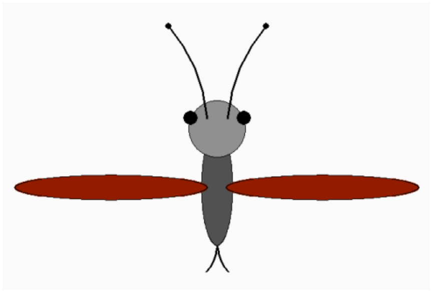

| |
|---|
| Cim Fea |
| Kei Fea |
| Sko Dav |
| Lif Dav |

Figure A2. The *subordinate-level without comparison* condition in Experiment 1

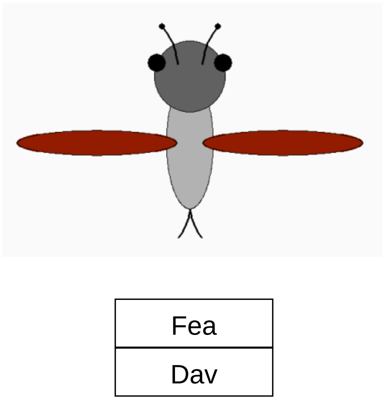


Figure A3. The *basic baseline* condition in Experiment 1

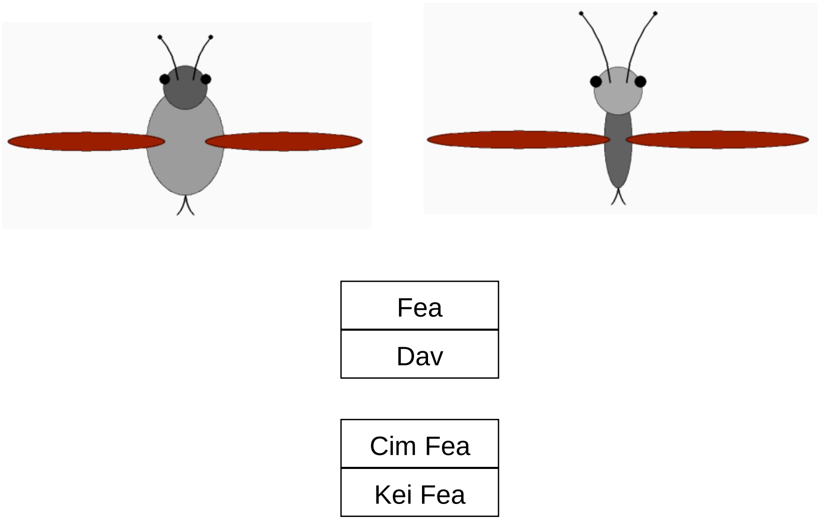


Figure A4. The *basic-level first with comparison* condition in Experiment 2. The pair shows Fea species (basic-level) and Kei Fea (subordinate-level).

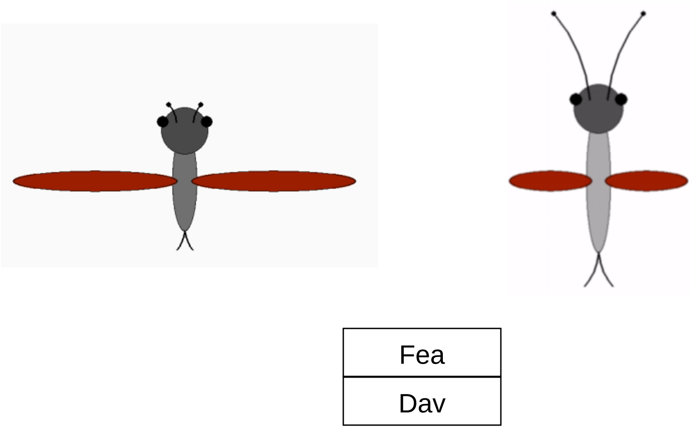


Figure A5. The *basic-level only with comparison* condition in Experiment 2.

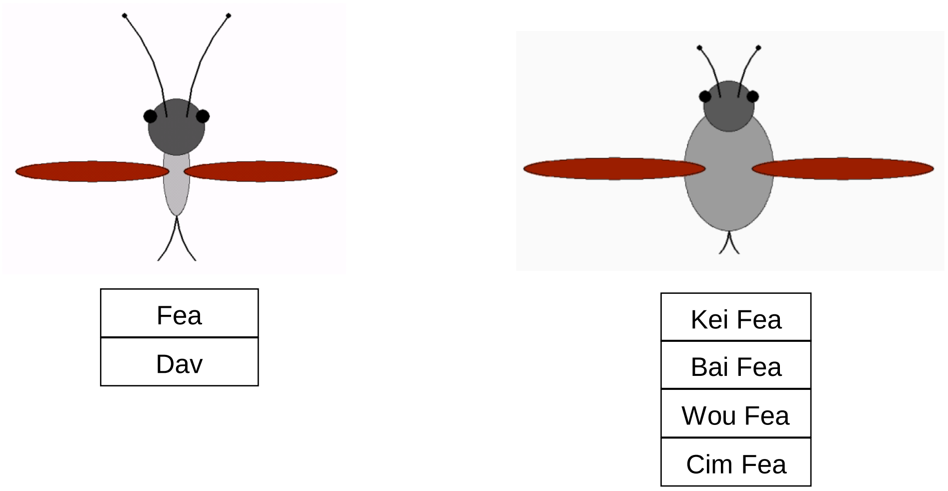


Figure A6. The *prototype* condition in Experiment 3. The bug in the left side is the prototype, and the right one is the exemplar.

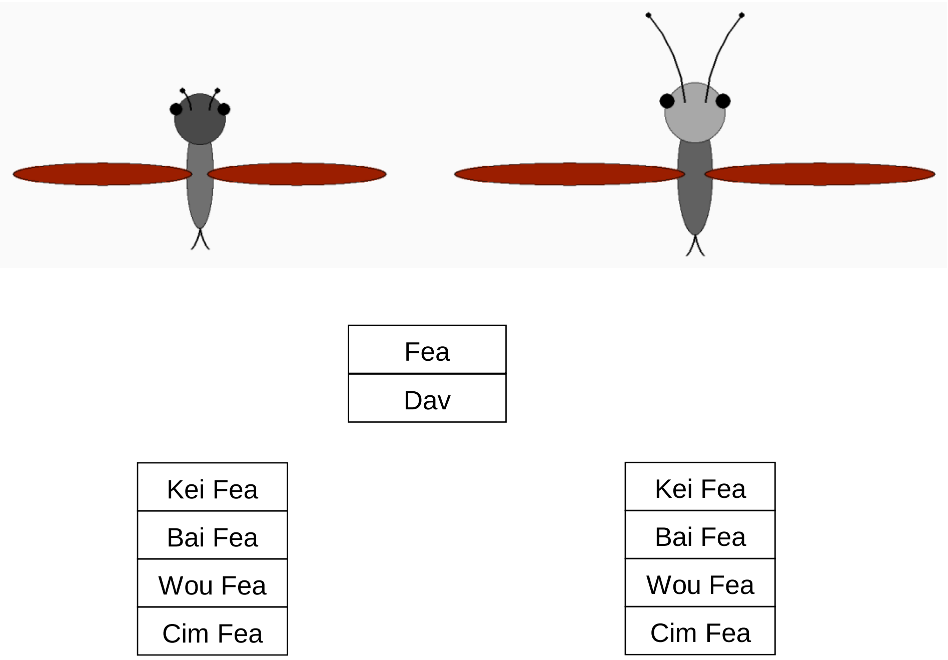


Figure A7. The *two different exemplars* condition in Experiment 2. They belong to the Fea species in the basic-level and to Wou Fea and Bai Fea in the subordinate-level, respectively.

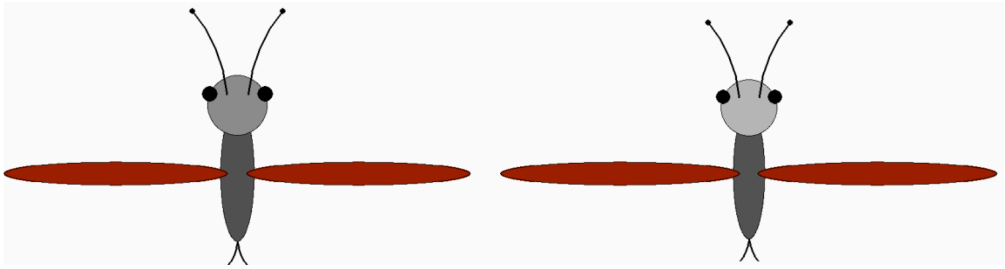

| |
|---|
| Fea |
| Dav |

| |
|---|
| Kei Fea |
| Bai Fea |
| Wou Fea |
| Cim Fea |

Figure A8. The *two same exemplars* condition in Experiment 2. Two bugs belong to the Fea species in the basic-level and Bai Fea in the subordinate-level.

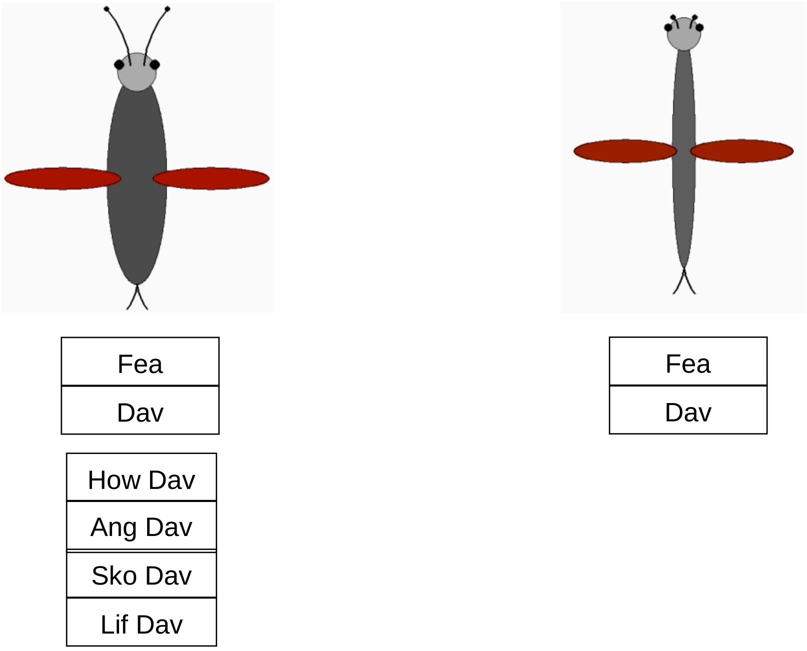


Figure A9. The *subordinate baseline* condition and *basic baseline* condition in Experiment 3, respectively.
